# Teeth for the Old

**Published:** 1861-09

**Authors:** 


### TEETH FOR THE OLD.

Deserved eminence in any calling can be attained only by years of patient study and unremitting toil. We have known a man for over fifteen years who for all their summers has bent over the fiery furnace heated to whiteness, and for many long winter nights retired not to his rest until the small hours of the morning came, only to rise again as soon as there was daylight to work by, spending money the meanwhile by tens of thousands, to find himself poor at last. This man is Professor Allen, of Bond-street, New York, who occupied at one time the highest chair in the Dental College of Ohio, president of the late Dental Convention of New York, and the industrious and able correspondent of the principal dental journals of the United States. To professional ability there is added a disposition too generous and too kind to escape impositions and injustices enough to quench all human sympathy, and yet he bears on his face the very impress of unsuspicious benevolence and unclouded hopefulness of a fruitful future.

It is almost incredible, but it is true, as we know for ourself, that now, for a week at a time, he scarcely sleeps four hours in the twenty-four, in the admiration he has for his improvements, and the wish to fill the urgent calls for immediate work which press upon him; for there are met in his office men who are themselves eminent in the dental profession by means of a devoted service to it for twenty and thirty years, and who have come to him for themselves or families, whose names and residences we know.

These statements are made more specially in reference to the invaluable benefits which Dr. Allen's improvements are able to confer on clergymen whose enunciation is imperfect from loss of teeth, and for those who, by reason of their cheeks falling in, look prematurely old. The difference in Dr. Allen's own appearance, when his improvements are in and out, is simply and unexaggeratedly wonderful. Clergymen whose legitimate profession is their only source of income are charged very little beyond the actual cost of the materials used and the time expended on them. Those who are in good circumstances are expected to pay liberally. Visitors are shown the photographs of persons whose teeth, gums, or cheeks are defective, and of the same after Allen's adjustments have been made. The inspection of these cannot but fill the beholder with admiration, as much at the perfection of the work as of the genius and indomitable perseverance of the man whose skill has accomplished it all; and we are highly gratified to know that he is beginning to reap a rich pecuniary harvest from the work of his hands, and quite as much so that there is no reasonable prospect of his professional services being required for our single self or double self for many a long, long year to come. To those of our readers who are curious to know what human art can accomplish in the way of rejuvenating the cheeks, and of making whole sets of teeth and gums in one solid piece, and so near nature in color, tint, and beauty, a visit to the Professor's rooms, in Bond-street, will be attended with the highest satisfaction.

